# Auditory and Visual Memories in PTSD Patients Targeted with Eye Movements and Counting: The Effect of Modality-Specific Loading of Working Memory

**DOI:** 10.3389/fpsyg.2017.01937

**Published:** 2017-11-03

**Authors:** Suzy J. M. A. Matthijssen, Liselotte C. M. Verhoeven, Marcel A. van den Hout, Ivo Heitland

**Affiliations:** ^1^Altrecht Academic Anxiety Center, Altrecht GGz, Utrecht, Netherlands; ^2^Department of Clinical Psychology, Utrecht University, Utrecht, Netherlands; ^3^GGz Centraal, Amersfoort, Netherlands; ^4^Symfora Meander, Amersfoort, Netherlands; ^5^Hannover Medical School, Hanover, Germany

**Keywords:** EMDR, working memory taxation, visual intrusions, auditory intrusions, modality specificity, eye movements

## Abstract

**Introduction:** Eye movement desensitization and reprocessing (EMDR) therapy is an evidence-based treatment for post-traumatic stress disorder (PTSD). A key element of this therapy is simultaneously recalling an emotionally disturbing memory and performing a dual task that loads working memory. Memories targeted with this therapy are mainly visual, though there is some evidence that auditory memories can also be targeted.

**Objective:** The present study tested whether auditory memories can be targeted with EMDR in PTSD patients. A second objective was to test whether taxing the patient (performing a dual task while recalling a memory) in a modality specific way (auditory demanding for auditory memories and visually demanding for visual memories) was more effective in reducing the emotionality experienced than taxing in cross-modality.

**Methods:** Thirty-six patients diagnosed with PTSD were asked to recall two disturbing memories, one mainly visual, the other one mainly auditory. They rated the emotionality of the memories before being exposed to any condition. Both memories were then recalled under three alternating conditions [visual taxation, auditory taxation, and a control condition (CC), which comprised staring a non-moving dot] – counterbalanced in order – and patients rerated emotionality after each condition.

**Results:** All three conditions were equally effective in reducing the emotionality of the auditory memory. Auditory loading was more effective in reducing the emotionality in the visual intrusion than the CC, but did not differ from the visual load.

**Conclusion:** Auditory and visual aversive memories were less emotional after working memory taxation (WMT). This has some clinical implications for EMDR therapy, where mainly visual intrusions are targeted. In this study, there was no benefit of modality specificity. Further fundamental research should be conducted to specify the best protocol for WMT.

## Introduction

Post-traumatic stress disorder (PTSD) is a debilitating disorder which is categorized as a trauma- and stressor-related disorder in DSM 5. It can be developed after being exposed to a traumatic event. The disorder is characterized by suffering from repeatedly re-experiencing the traumatic event (in flashbacks or nightmares), avoidance of trauma-related stimuli, negative alterations in mood and cognition, and alterations in arousal and reactivity (American Psychiatric Association, 2015). Several psychological treatments are effective in treating PTSD. One of those treatments is eye movement desensitization and reprocessing (EMDR) therapy. A core feature of EMDR therapy is that a disturbing memory is held in mind by a patient while simultaneously making horizontal eye movements (EMs). These movements are typically induced by following a moving dot that is displayed on a light bar or the therapist’s fingers, moving a hand continuously back and forth in front of the patient’s eyes. Clinical trials and meta-analyses have demonstrated the effectiveness of EMDR in treating PTSD (for meta-analyses, see, e.g., Bradley et al., 2005; Seidler and Wagner, 2006; Bisson et al., 2007; Chen et al., 2014; Cusack et al., 2016).

Evidence that EMDR is an effective treatment for PTSD does not imply knowing what the underlying working mechanism is. One explanatory hypothesis for how EMDR works, which is gaining accumulating evidence, is based on the working memory (WM) model (Baddeley and Hitch, 1974). The hypothesis states that recalling memories requires WM resources, which are limited. If a dual task, which also uses WM capacity, is performed during recall, fewer resources will be available for recall. As a consequence, the recalled memory will be less emotional and less vivid and will be reconsolidated as less emotional and less vivid in long-term memory (Van den Hout et al., 2010). EMs are considered a dual task. Consistent with the hypotheses from WM theory, memories have been found to not only become less disturbing and less vivid after execution of an EM task but also after a range of other tasks that load WM (e.g., counting, watching an array of small squares that constantly and randomly change between black and white, mindful breathing) (e.g., Andrade et al., 1997; Kavanagh et al., 2001; Kemps and Tiggemann, 2007; Gunter and Bodner, 2008; Van den Hout et al., 2010, 2011a; Engelhard et al., 2011).

In therapy, EMDR focuses on the intrusive memories of traumatic events – one of the hallmark symptoms of PTSD. Ehlers et al. (2002) asked patients with PTSD to describe the content of their typical intrusive memory and concluded that visual intrusions were more common (70–97%) than bodily sensations (28–66%), sounds (38–51%), smell (48–51%), actions (22–65%), or thoughts (26–60%). Hackmann et al. (2004) interviewed 22 patients with chronic PTSD about the content of their intrusive memories and found the majority included visual and/or bodily sensations. Auditory content was experienced in about half of the intrusions. Taste and smell sensations were least common. Hence, it is clear that intrusive memories can appear in different sensory modalities. EMDR aims at reducing PTSD symptoms by reducing emotional intensity of *visual* images. However, the question remains if intrusions in other sensory modalities can be successfully targeted with EMDR?

The WM model (Baddeley and Hitch, 1974) comprises the central executive (CE) and two so-called “slave” systems; the visuospatial sketchpad (VSSP) and the phonological loop (PL). The CE carries out higher order cognitive functions (i.e., problem solving and planning), whereas the VSSP is concerned with processing and storing visual and spatial information and the PL with processing and storing auditory information (Andrade et al., 1997). The VSSP is thus involved in visual imagery and the PL in auditory imagery (Kristjánsdóttir and Lee, 2011). Earlier studies show some inconsistencies in whether the CE is merely responsible for the reduction in vividness and emotionality of memories or if this is a consequence of loading the slave systems, the latter implying a benefit of modality-specific demanding tasks (Andrade and Baddeley, 1993 in Andrade et al., 1997; Baddeley and Andrade, 2000; Gunter and Bodner, 2008; Kristjánsdóttir and Lee, 2011). In a series of experiments Andrade and Baddeley (1993 in Andrade et al., 1997) showed that counting made auditory images less vivid, whereas tapping tasks made visual images less vivid. They asked participants to imagine how things looked or sounded. They did so while performing either a task taxing the PL (counting) or the VSSP (tapping a pattern). After imagining how things looked or sounded they were asked to rate the vividness of their image on a scale from 0 (no image) to 10 (as clear as normal). Tasks matched in modality appeared to have a larger effect on vividness ratings than tasks not matched in modality. Andrade et al. (1997) conducted another series of experiments where they asked participants to imagine neutral or negative stimuli (consisting of earlier presented neutral or negative photographs) and to perform different dual tasks (counting, a simple tapping task, a complex tapping task, and EM) and a control task (monitoring a non-moving letter on a screen). They consistently found concurrent tasks had a larger effect on vividness. The results were less clear and less consistent for emotionality. In the last of their series of experiments they used personal memories and found that concurrent visuospatial tasks reduced the emotionality ratings, but the effect was much smaller for the vividness ratings. They concluded that the locus of the effect was the VSSP (Andrade et al., 1997). However, the authors did not test the effect of a concurrent phonological load on auditory personal memories. Baddeley and Andrade (2000) conducted seven experiments, exposing participants to novel stimuli, being either visual or auditory (e.g., shapes or musical notes) while conducting a visual, auditory, or control dual task. They found an interaction between modality of images and the dual task on vividness ratings. For familiar or meaningful scenes or sounds this modality-specific effect was still present, but smaller. Baddeley and Andrade (2000) therefore concluded that the slave systems are involved in reducing vividness, and that the CE also plays a role here.

A limitation of the studies described above is that there were no baseline measurements. Participants rated their images after the working memory taxation (WMT), leaving it unclear if there was any difference before conducting the task. Kemps and Tiggemann (2007) conducted two studies to investigate the effect of concurrent visual and auditory interference on emotional images, one of them contained a baseline measurement. They instructed 68 undergraduates to recall a specific visual or auditory image of happy and distressing memories, while they were exposed to either EM, articulatory suppression (counting aloud), or a control condition (CC). There was a large general effect of WM loading, but superimposed on that general effect, the authors reported a modality-specific effect: vividness and emotionality ratings were reduced to a greater extent when the modality of taxation was matched to the modality of the image.

Gunter and Bodner (2008), however, found no effect of modality specificity in reducing the distress of negative memories. They asked participants to hold distressing memories in mind while performing an auditory shadowing task or a demanding visuospatial task or EM. They found equal benefits for EM and the auditory task, but a demanding visuospatial task was more beneficial. Furthermore, Kristjánsdóttir and Lee (2011) asked participants to recall an unpleasant autobiographical memory while performing each of three dual-attention tasks (EM, listening to counting, or a CC). They found that EM led to a greater decrease in vividness than listening to counting. They also found that EM and listening to counting were equally effective in reducing emotionality. Both effects were present irrespective of the modality of the memory. This was taken to support the crucial role of the CE relative to the VSSP or the PL. However, it is unclear how cognitively demanding the tasks were, leaving it unclear if effects could really be attributed to CE or if the VSSP and PL still play a role.

The studies reported by Gunter and Bodner (2008) and by Kristjánsdóttir and Lee (2011) were carried out to clarify how EMDR yields it positive effects. A crucial limitation of their studies is that non-clinical samples were used and, therefore, it is unclear whether the findings can be generalized to PTSD patients. The issue is an empirical one. Given its clinical importance it requires settling, although there may be no reason in advance to believe that a clinical sample would react differently than a non-clinical sample to WMT on disturbing memories. A second, perhaps more important limitation is that none of the studies cited above actually measured the *degree* of WMT of the dual tasks being used. This can lead to the conclusion – if not finding a modality specific effect – that the effect can be attributed to the CE, while it could actually be a consequence of a task being more demanding than another task. Also, no modality specificity can be inferred if the analysis only includes visual memories, hence a dual visuospatial task could just require more effort than a dual auditory task. A model in which both the CE and the slave systems are responsible for the effect on emotionality and vividness in emotional disturbing images is also possible. This would therefore lead to an absence of the modality specificity effect found in some of the previous studies.

In summary, some of the above studies indicate that auditory memories can be made less emotional and vivid by dual tasks in non-clinical samples. Furthermore, there are some studies indicating there is a greater reduction of vividness and emotionality ratings if the dual task is matched to the modality of the memory. The aim of this study is to test whether auditory intrusions can be targeted with EMDR in PTSD patients. A second objective is to test whether modality-specific loading [auditory (visual) loading of auditory (visual) intrusions] is more effective in reducing the emotionality experienced than taxing in cross modality.

## Materials and Methods

### Patients

Thirty-eight patients with PTSD were recruited to the study. Diagnosis of PTSD was made by a trained clinician (clinical psychologist/psychiatrist) and based on DSM IV-TR criteria (American Psychiatric Association, 2000). Two patients were excluded on starting participation. One was too scared to participate and expressed that she thought she was unsuitable for the experiment. The other patient was unable to select memories which could be targeted. Data from 36 patients (32 females and 4 males) with a mean age of 39.19 (*SD* = 11.19) were collected. Apart from the PTSD, 77.8% had at least one other Axis I diagnosis and 33.3% had at least one Axis II diagnosis. They all received treatment in several Dutch mental health institutions. Eighteen patients received treatment at an Academic Anxiety Center, nine at a Medical Center, and nine at different Faculty Assertive Community Treatment Centers. Apart from being diagnosed with PTSD, inclusion criteria were that the patient had to have an estimated IQ higher than 80, be at least 18 years of age and have sufficient mastery of the Dutch language. Exclusion criteria were an acute suicide risk and severe visual or hearing impairments. IQ, mastery of the Dutch language, and suicide risk were estimated by the therapist referring the patient for the study. No data were obtained about the type of trauma, length or quantity of the trauma, or years since index trauma. Therefore, no exclusions were made based on one of these trauma-related factors. Although data from 36 patients were collected, for the auditory memory, data from only 30 patients (*M* = 38.93, *SD* = 12.09) were included into the analysis and for the visual memory this was the case for 31 patients (*M* = 39.58, *SD* = 12.09). (See design for further explanation on this.) For specific patient characteristics see **Table 1**.

**Table 1:** Patient characteristics.

	Auditory memory	Visual memory
	(*N* = 30)	(*N* = 31)
**Gender**		
Female	26 (86.7%)	27 (87.1%)
Male	4 (13.3%)	4 (12.9%)
**Axis I disorder**		
PTSD	7 (23.3%)	7 (22.6%)
PTSD + mood disorder	9 (30%)	7 (22.6%)
PTSD + anxiety disorder	5 (16.7%)	7 (22.6%)
PTSD + other disorders	6 (20%)	7 (22.6%)
PTSD + addiction + other	2 (6.7%)	2 (6.5%)
PTSD + addiction	1 (3.3%)	1 (3.2%)
**Comorbid Axis II disorder**		
No diagnosis	19 (63.3%)	23 (74.2%)
≥Axis II diagnosis	11 (36.7%)	8 (25.8%)
**Education level**		
Primary school	2 (6.7%)	2 (6.5%)
Secondary school	11 (36.6%)	12 (38.7%)
Lower vocational education	1 (3.3%)	1 (3.2%)
Secondary vocational education	10 (33.3%)	9 (29%)
Higher professional education	6 (20%)	7 (22.6%)
**Psychopharmacological drugs**		
No use of medication	6 (20%)	7 (22.6%)
Antidepressants (AD)	7 (23.3%)	6 (19.4%)
Benzodiazepines (BD)	1 (3.3%)	3 (9.7%)
Antipsychotics (AP)	1 (3.3%)	1 (3.2%)
AD and/or BD and/or AP	5 (16.5%)	5 (16%)
Other (single or combination)	10 (33.3%)	9 (28.8%)

### Procedure

Study procedures were approved by the medical ethics institutional review board of the University Medical Center, Utrecht, Netherlands. Therapists from the participating mental health institutions were asked to check their caseload, select all patients meeting the criteria, and approach them for participation. Patients were given an information letter and were able to consider participating for at least a few days. Upon giving oral consent to their therapist they were referred to the researchers. The researchers are unaware whether and how many patients refused participation. All patients received treatment as usual while participating in the study.

After giving written informed consent, patients were briefed in short about the study. They were instructed to recall two emotionally disturbing memories that were still giving emotional distress, one mainly auditory and one mainly visual. While recalling the visual (auditory) memory, the subjects were instructed to either consequently make EM (visual taxation, VT), to count down (auditory taxation, AT) or to stare at a non-moving dot (CC). After selection, the extent to which the memories were auditory or visual was rated on one 100 mm Visual Analog Scale (VAS), ranging from completely auditory to completely visual. For selection, a threshold of 50% auditory (visual) was applied. After this, other sensory modalities (gustatory, kinesthetic, and olfactory) were checked whether they were not more dominant than the auditory (visual) modality in the selected memory, by asking participants to divide a 100 mm VAS to the extent in which all sensory modalities were present in the memory. The order of the type of memory (visual vs. auditory) and the conditions (VT, AT, and CC) were counterbalanced. Once instructed, the patients were asked to recall the emotionally disturbing [visual (auditory)] memory and to rate the disturbance on a scale from 0 to 10 [the subjective units of disturbance (SUD) score; see below]. The memories were then recalled approximately 30 min each, while being exposed to each condition (VT, AT, and CC) twice for 5 min. To mimic EMDR procedures, after every 1 min during a 5-min period the condition was interrupted to check what was going through the patient’s mind. Answers were not discussed by their content but were followed by the instruction “concentrate on that” after which the next 1-min period of the condition was continued.

During each condition, participants were seated in front of a light bar. During the CC, the bar displayed a non-moving dot in the center of the bar. During the VT, a moving dot was displayed. During the AT, the bar displayed nothing. The speed used for the moving dot in the VT condition and the type of counting task was based on previous research from Van den Hout et al. (2010, 2011a) and Engelhard et al. (2011). In these studies individuals carried out a reaction time (RT) task. An increase in response time was observed when an additional task was added. The delay in response time as a result of EMs with 1 cycle (left–right–left) per second (RT of 115 ms) versus the response delay as a result of a countdown from 1000 (RT of 97 ms) was approximately equal (Engelhard et al., 2011; van den Hout et al., 2011b). Therefore, these two tasks were considered suitable to induce similar WM load.

### Design

**FIGURE 1:**
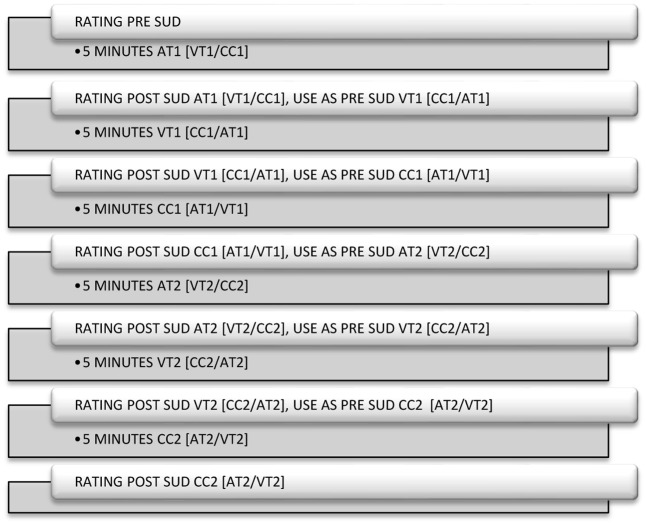
Timeline showing the presentation for all conditions.

The study had a two (time; pre- and post-) by three (conditions: VT, AT, and CC) repeated measures within-subject design. For a detailed timeline see **Figure 1**.

This design was used both for the auditory as well as the visual memory. The dependent variable was the SUD score, which indicated the level of distress or emotional disturbance experienced by the patient in terms of the recalled emotional target image. SUD scores were verbally expressed by the patient and SUD scores are routinely used in EMDR. Data were analyzed with SPSS version 23. To obtain sufficient statistical power (power 0.8, with an α-level of 0.05 and an expected medium effect size, *f* = 0.25), 36 patients were needed.

Although the intention was to present all conditions (VT, AT, and CC) twice, 6 out of 36 patients reached SUD 0 – meaning experiencing no emotional disturbance when recalling the auditory memory – before the presentation of all conditions was completed. Before completing all conditions twice, 21 patients reached SUD 0. Clinically, this was an encouraging observation demonstrating that this procedure was efficient in reducing SUD scores. As there was insufficient data for the second presentation, the respective SUD was excluded, meaning only data pertaining to the first exposure was analyzed. Hence, the final sample comprised 30 patients.

The same pattern of rapidly decreasing SUD was observed for the visual memory. Five out of 36 patients did not complete all conditions at least once, and in total only 14 patients were presented with all conditions twice. One person stopped halfway during the experiment because he was tired, but still was included into the analyses, because he went through all conditions once. Thirty-one patients were included in the analyses and their first exposure to the three conditions.

### Materials

#### Subjective Units of Disturbance (SUD)

Subjective units of disturbance scores ranged from 0 (no emotional disturbance) to 10 (the worst emotional disturbance possible). Patients were asked to verbally rate their SUD scores concerning the emotional target image before and after each condition (VT, AT, and CC).

#### EMDR Protocol

Patients were tested individually by the researchers (authors 1 and 2; both EMDR therapists) using steps 1, 2 and 3 (introduction, assessment, and desensitization) from the standard Dutch EMDR protocol (De Jongh and Ten Broeke, 2012). A slightly altered version was used for the auditory memory. In this altered version, all words referring to “visual” sensory modality were altered into words referring to the auditory modality.

## Results

### Baseline

The average SUD pre-score was 8.97 (standard deviation, *SD* = 0.96) for the auditory memory (*N* = 30) and 8.87 (*SD* = 1.06) for the visual memory (*N* = 31). The difference was not significant [*t*(35) = 0.19, *p* = 0.85].

### Auditory Memory

**FIGURE 2:**
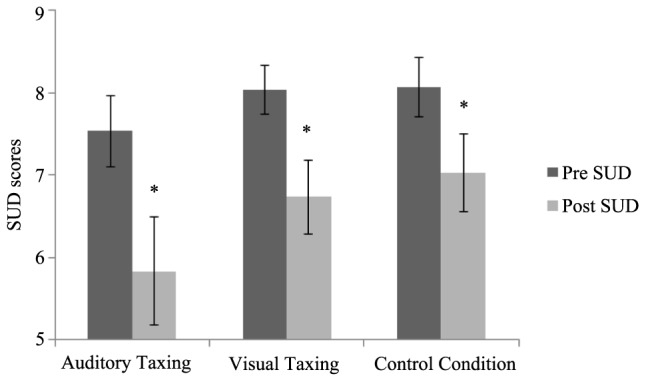
Pre- and post-SUD scores of the auditory memory are shown per condition. Error bars depict ±1 SEM (^∗^*p* < 0.05).

A two (time: pre- and post-) by three (conditions: VT, AT, and CC) repeated measures ANOVA was conducted. A main effect for time [*F*(1,29) = 42.00, *p* < 0.01] was found, but there was no main effect for condition [*F*(2,58) = 2.02, *p* = 0.14] and no time × condition interaction [*F*(2,58) = 1.70, *p* = 0.19] was found. The pre- and post-SUD scores of the VT, AT, and CC are depicted in **Figure 2**, showing that regardless of the condition, the SUD dropped from pre- to post.

### Visual Memory

**FIGURE 3:**
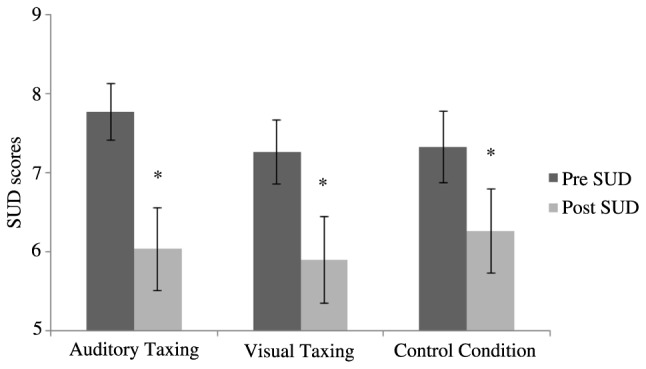
Pre- and post-SUD scores of the visual memory are shown per condition. Error bars depict ±1 SEM (^∗^*p* < 0.05).

A two (time: pre- and post-) by three (conditions: VT, AT, and CC) repeated measures ANOVA was conducted. The pre- and post-SUD scores of the VT, AT, and CC are graphically depicted in **Figure 3**, showing that, regardless of the condition, the SUD dropped from pre- to post. This was reflected in a main effect for time [*F*(1,30) = 47.06, *p* < 0.01]. There was no main effect for condition [*F*(2,60) = 0.25, *p* = 0.78]. However, a time × condition interaction [*F*(2,60) = 3.31, *p* = 0.04] was found. *Post hoc* analyses with no correction for multiple comparisons revealed AT outperformed the CC (*p* = 0.02) but none of the interactions differed significantly after Bonferroni correction was applied (*p* > 0.055).

## Discussion

The first aim of the present study was to test whether auditory intrusions could be successfully targeted with EMDR in PTSD patients. The second aim was to assess whether modality-specific loading of WM was more effective than providing non-modality-specific loads in reducing emotionality experienced in auditory and visual intrusions. This was assessed by asking PTSD patients to recall an auditory and visual emotional memory while engaging in modality-specific WMT (EMs or counting) or a CC. Although earlier studies showed the effect of WMT on non-autobiographical auditory material (e.g., Andrade et al., 1997; Baddeley and Andrade, 2000) and on autobiographical memories with (some) auditory content (Kemps and Tiggemann, 2007; Kristjánsdóttir and Lee, 2011) in non-clinical samples, to the best of our knowledge this is the first study to examine this in patients. Earlier studies did not control for the degree of interference of the tasks on the WM. The current study did try to match the degree of loading in the relevant condition (EMs and counting) in an attempt to improve the comparison. The results of the study are clear and indicate that emotionality can be reduced in both visual and auditory disturbing memories in PTSD patients. Furthermore, no difference was found between AT, VT, or the CC. This indicates no modality-specific effect and no support for the efficacy of WMT.

A possible explanation for finding an effect in the CC is that the CC may also be demanding. Although Lee and Cuijpers (2013) showed an additive effect of EMs in EMDR treatment and laboratory studies [significantly moderate (Cohen’s *d* = 0.41) and significantly large (*d* = 0.74)], this was not found in a recent study by Sack et al. (2016). They found EMs had no advantage over fixation on a non-moving hand. Our hypothesis is that fixation on a non-moving stimulus still requires cognitive resources. This was also strengthened by the observation by the researchers that some patients in the CC were intensely focused on the non-moving dot. However, future research should address whether staring at a non-moving dot also requires effort or if there is another explanation for the absence of difference in effect between the AT and VT versus the CC. A possible explanation for not finding a modality-specific effect is that – although the tasks were specifically chosen to be equally demanding – the tasks may actually not have been exactly matched and possibly the auditory dual task was more taxing than the visual task. On the other hand, some patients had difficulty pursuing the moving dot and were therefore unable to follow it at times. This could potentially have led to missing out on WMT. It is also possible that the auditory and visual tasks are not equally loading the PL or the VSSP, respectively, but that the AT has a more cognitive component to it than the VT, hence using more of the CE capacity. Furthermore, there can be individual differences in PL and VSSP functioning, which were not taken into account. Furthermore, the CC may have a more cognitive component than the VT or a more visual component than the AT. Future research should therefore address these points and could pre-test individuals with a RT test to optimize the comparability of the tasks.

A limitation of the study is the sample size. The power calculation showed 36 patients needed be included, whereas only 30 and 31, respectively, were included for analyses of the auditory and visual memory. The other patients had already reached SUD 0 (meaning experiencing no emotional distress) exposure to all conditions. This being a very welcome observation on the one hand, creates a power-problem on the other hand.

Working with visually disturbing memories in EMDR therapy does elicit positive effects on PTSD symptoms, so it is expected that this effect is generalizable to memories in other sensory modalities. Although future research is needed to examine whether EMDR or staring at a non-moving dot (the CC) for emotionally disturbing auditory memories has an effect on PTSD symptoms, positive clinical effects may be anticipated. The current study only consisted of one experimental “session” and no symptoms of PTSD were measured. Measuring the severity of PTSD symptoms and offering multiple sessions to patients are recommended for future research.

## Ethics Statement

This study was carried out in accordance with the recommendations of “Medical Ethical Committee of the University Medical Center, Utrecht” with written informed consent from all subjects. All subjects gave written informed consent in accordance with the Declaration of Helsinki. The protocol was approved by the “Medical Ethical Committee of the University Medical Center, Utrecht.”

## Author Contributions

SM, LV, and MvdH designed the research. SM and LV collected the data. SM, LV, and IH analyzed the data. SM, MvdH, IH, and LV wrote the paper and approved the final manuscript.

## Conflict of Interest Statement

The authors declare that the research was conducted in the absence of any commercial or financial relationships that could be construed as a potential conflict of interest.
